# Formation of a Trimeric Xpo1-Ran[GTP]-Ded1 Exportin Complex Modulates ATPase and Helicase Activities of Ded1

**DOI:** 10.1371/journal.pone.0131690

**Published:** 2015-06-29

**Authors:** Glenn Hauk, Gregory D. Bowman

**Affiliations:** T.C. Jenkins Department of Biophysics, Johns Hopkins University, Baltimore, Maryland, United States of America; University of Toronto, CANADA

## Abstract

The DEAD-box RNA helicase Ded1, which is essential in yeast and known as DDX3 in humans, shuttles between the nucleus and cytoplasm and takes part in several basic processes including RNA processing and translation. A key interacting partner of Ded1 is the exportin Xpo1, which together with the GTP-bound state of the small GTPase Ran, facilitates unidirectional transport of Ded1 out of the nucleus. Here we demonstrate that Xpo1 and Ran[GTP] together reduce the RNA-stimulated ATPase and helicase activities of Ded1. Binding and inhibition of Ded1 by Xpo1 depend on the affinity of the Ded1 nuclear export sequence (NES) for Xpo1 and the presence of Ran[GTP]. Association with Xpo1/Ran[GTP] reduces RNA-stimulated ATPase activity of Ded1 by increasing the apparent KM for the RNA substrate. Despite the increased KM, the Ded1:Xpo1:Ran[GTP] ternary complex retains the ability to bind single stranded RNA, suggesting that Xpo1/Ran[GTP] may modulate the substrate specificity of Ded1. These results demonstrate that, in addition to transport, exportins such as Xpo1 also have the capability to alter enzymatic activities of their cargo.

## Introduction

Ded1 and its orthologs have been implicated in a variety of nuclear and cytoplasmic processes, from splicing to translation. Although essential for growth in yeast, the substrate specificities of Ded1 and the effects of binding partners on Ded1 activities have not been fully explored. In the cytoplasm, Ded1 has been shown to be required as a translation initiation factor [1, 2]. Studies from yeast have shown that Ded1 functions as an assembly point for an mRNA-protein complex containing eIF4E/eIF4G, which accumulates in sub-cellular bodies containing non-translating ribonucleoprotein (RNP) complexes called stress granules [3]. Ded1 facilitates the exit of RNPs from stress granules in an ATPase-dependent manner [3]. Ded1 is therefore positioned at a key regulatory stage, where assembly of Ded1-containing complexes inhibits translation, and ATPase activity of Ded1 is required for the progression of messenger RNPs (mRNPs) to translation initiation.

In the nucleus, the role of Ded1 and its orthologs is less clear, but genetic [4] and biochemical [5, 6] data point to associations with splicing machinery. Recently, Senissar and colleagues demonstrated that Ded1 is physically associated with nuclear and cytoplasmic assemblies that bind the 5’methylguanosine cap of mRNAs [7]. Ded1 was shown to shuttle between the nucleus and cytoplasm in yeast *via* canonical mRNA export pathways that utilize mex67 and through protein export pathways using the exportin Xpo1 [7]. These findings are consistent with the observation that Ded1 orthologs from *Xenopus* (An3) and human (DDX3X) shuttle from the nucleus to the cytoplasm through the Xpo1/CRM1 adaptor [8, 9].

For nuclear trafficking, exportins such as Xpo1 utilize the GTP-bound state of the small GTPase Ran (Ran[GTP]) as a cofactor for cargo binding [10, 11]. The nucleus contains the chromatin bound nucleotide exchange factor RCC1, which facilitates the charging of Ran with GTP, while the cytoplasm contains factors that stimulate hydrolysis of GTP by Ran. This partitioning of factors that bias the nucleotide-bound states of Ran into distinct cellular compartments establishes a Ran[GTP] gradient: high concentrations of Ran[GTP] are maintained in the nucleus, where exportin-cargo binding occurs, whereas Ran[GTP] becomes depleted in the cytoplasm, which along with other factors, aids disassembly of exportin complexes. Xpo1 is responsible for maintaining nucleo-cytoplasmic distributions of a variety of proteins, including snurportin 1 (SPN1), which shuttles between the nucleus and the cytoplasm and is responsible for the nuclear import of cytoplasmically processed small nuclear RNA protein complexes (snRNPs). Xpo1 is responsible for the re-export of snurportin 1 to the cytoplasm, and snurportin 1 has been used as a model for Xpo1:cargo interactions. Structural characterization of Xpo1 in complex snurportin 1 revealed that snurportin binds at the outer rim of a toroidally shaped Xpo1 molecule, with Ran[GTP] binding in the central “donut hole” of the toroid [12, 13].

The *Xenopus laevis* Ded1 ortholog (An3) has been shown to interact with Xpo1, dependent on Ran[GTP] and the An3 nuclear export sequence (NES) [8, 14]. Additionally, the removal of the first 30 amino-terminal residues of Ded1, including the NES results in nuclear accumulation in yeast [7]. Although the NES at the extreme N-terminus of Ded1 orthologs is conserved from yeast to humans, the human ortholog of Ded1 (DDX3) was found to bind to the Xpo1 ortholog CRM1 independently of Ran[GTP] and its NES-containing N-terminus [9]. These results were used to argue that human DDX3 interacts with CRM1 distinctly from other typical cargo of Xpo1.

Helicase-type ATPases similar to Ded1 are commonly found to be regulated by protein segments outside the core motor [15]. Given the multiple binding modes reported for Ded1 orthologs and importance of ATPase regulation, we were interested to explore Ded1 binding to Xpo1 and possible effects on Ded1 activity. Here we report that stable binding of Ded1 to Xpo1 requires Ran[GTP] and an intact Ded1 nuclear export sequence. Small-angle X-ray scattering (SAXS) data suggest that Ded1, like snurportin 1, interacts at the edge of a toroidally shaped Xpo1 molecule, which is distinct from the central cargo-binding location of other exportins. The Ran[GTP]- and NES-dependent interactions of Ded1 with Xpo1 increase the apparent K_M_ for Ded1 activity on yeast whole cell RNA, resulting in reduced ATPase activity at low concentrations of a mixed RNA substrate. Ternary complex formation also lowers overall helicase activity of Ded1 on a defined RNA substrate. While catalytic activity of Ded1 was reduced by binding to Xpo1 and Ran[GTP], we find that Ded1 remains competent for the formation of stable, ATP-dependent complexes with single-stranded RNA (ssRNA), suggesting that RNA binding surfaces of Ded1 are not occluded. These results reveal that catalytic activities of a DEAD-box helicase can be modulated upon forming an exportin complex, which may be used to redirect Ded1 specificity in the context of an Xpo1 export complex.

## Materials and Methods

### Cloning and mutagenesis

Full-length Xpo1 (1–1071), full-length Ded1 (1–604), Ded1ΔC (1–544), Ded1NTD (1–368), and full-length Ran (1–219) were PCR amplified from *S*. *cerevisiae* genomic DNA. Amplified fragments of Ded1 and Ran were cloned into a modified pET28 vector containing a preScission protease site upstream of the multiple cloning site; Xpo1 was sub-cloned into a His-tagged pGEX vector using standard protocols. Modified versions of Ded1 (I13A and sNES) and Ran_Q71L_ were generated using a dual single-primer mutagenesis strategy [16]. Primers for the sNES variants were designed based on the NES determinants outlined by Güttler *et al*. [17], which changed the natural NES sequence of Ded1 **GP**MAELSEQVQNLSI to **GP** DMDELSKKFGTLSI (residues modified or inserted to improve affinity are underlined, and those from the pET28 vector are bold).

### Protein expression and purification

All constructs were expressed in BL21(DE3)(star) cells containing the Rosetta2 plasmid (Novagen) using the auto-induction method [18]. For Ded1 and Ran constructs, cells were grown at 37°C to an OD_600_ of 0.7, and then the temperature was reduced to 18°C for an additional 18–22 hours, after which the cells were harvested and stored at -80°C. Xpo1 failed to show significant expression using the T7-driven pET28 vector, and instead was expressed using a pGEX vector with a tac promoter, modified to include a hexahistadine tag upstream of the multiple cloning site. For Xpo1, cells were grown to an OD_600_ of 1.0 to 1.2 at 37°C, after which the temperature was reduced to 25°C for an additional 44–48 hr before cell harvesting.

After resuspension in lysis buffer (40 mM Tris pH 7.8, 1 M NaCl, 50 mM L-arginine, 50 mM L-glutamic Acid, 10 mM imidazole and 0.5mM TCEP pH 7.0), cells were lysed using lysozyme and sonication. The lysate was clarified by centrifugation and the His-tagged proteins were purified by Ni-affinity chromatography. After desalting into lysis buffer containing 15 mM imidazole, His-tags were removed by treatment with preScission protease, and then further purified by a second passage over a HisTrap column. Proteins were then concentrated and applied to a Superdex 200 size exclusion chromatography column as a final step. The final buffer for Xpo1 and Ran proteins was 20 mM Tris (pH 7.8) and 300 mM NaCl. Due to poor stability in low salt, the final buffer for Ded1 proteins contained 1 M NaCl. For Ran[GTP]_Q71L_, all buffers additionally contained 100 μM GTP (pH 7–8) and 1mM MgCl_2_, and 1 mM GTP was added to final Ran[GTP]_Q71L_ fractions. Protein concentrations were determined by A_280_ and by Bradford assay.

### Size exclusion chromatography

For all size exclusion experiments, a Superdex 200 10/300 column (GE Healthcare) was used, pre-equilibrated in gel filtration buffer (20 mM Tris pH 7.8, 300 mM NaCl, 100 μM GTP, 1 mM MgCl_2_ and 0.5mM TCEP). Xpo1 (10 μM), Ded1ΔC or Ded1ΔC variants (11 μM), and Ran[GTP]_Q71L_ (11 μM) were run separately or in the combinations indicated, using a 500μL injection volume. Fractions were collected in 1 mL aliquots, and analyzed on 15% SDS-PAGE gels stained with GelCode Blue Safe Protein Stain (Pierce). Chromatograms and gels are representative of 3 or more experiments for each sample.

### SAXS analysis

Samples for SAXS analysis were prepared as described for the size exclusion chromatography experiments above, except that higher protein concentrations were used: 52 μM Xpo1, 57 μM Ded1ΔC, and 68 μM Ran[GTP]_Q71L_. Fractions that contained the Xpo1-Ded1ΔC-Ran[GTP]_Q71L_ complex were conservatively pooled and concentrated to ~13 mg/mL. Gel filtration buffer was used to dilute pooled fractions for SAXS experiments. Data were collected at the Cornell High Energy Synchrotron Source, at beamline F2. Analysis of raw scattering data was conducted using RAW [19] and Primus [20]. DAMMIF was used to calculate 15 molecular envelopes, which were averaged and filtered using the programs DAMAVER and DAMFILT [21].

### ATPase assays

ATP hydrolysis was monitored using a NADH-coupled assay in a microplate reader as previously described [22]. Reactions were conducted in 25 mM Tris (pH 7.8), 120 mM NaCl, 5.5 mM MgCl_2_, 1 mM DTT and initiated by the addition of 5 mM ATP (pH 7.0). Protein concentrations were 500 nM for full length Ded1 and Ded1 variants in all experiments. For the Xpo1/Ran[GTP] titration experiments, the concentrations of Xpo1 ranged from 0.5 μM to 11 μM, with the corresponding reactions containing 0.65 μM to 13 μM Ran[GTP]_Q71L_. Whole cell yeast RNA concentration for these Xpo1/Ran[GTP] titration experiments was 320 μg/mL. For RNA titration experiments used to calculate K_M_, concentrations of Xpo1 and Ran[GTP]_Q71L_ were 5.5μM and 6.5μM, respectively. For whole cell yeast RNA titration experiments, RNA concentations ranged from 750 μg/mL to 183 ng/mL in four-fold dilution steps. For the poly(A) and poly(U) titration experiments, substrate was purchased as the lyophilized product of polynucleotide phosphorylase action from Midland Certified Reagent Company (www.oligos.com). As with whole cell RNA substrate, seven RNA concentrations of poly(A) and poly(U) were used to calculate K_M_, ranging from 2.5 mg/mL to 610 ng/mL in four-fold dilution steps. Non-linear curve fitting, using the equation *v* = V_max_[S]/K_M_+[S], was conducted to calculate V_max_ and K_M_ values.

### RNA helicase assays

The RNA substrate for helicase unwinding experiments, designed following refs. [23, 24], consisted of an unlabeled 41mer (5’ CGA AAG CAC CGU AAA CGA AAA CUA GCA CCG UAA AGC AAG CU) annealed with a FAM-labeled 13mer (5’ CGU UUA CGG UGC U) in a 1.1:1 ratio in 10 mM Tris (pH 7.5), 50 mM NaCl, 1 mM EDTA by heating to 95°C for 3 minutes and cooling slowly to room temperature. Duplex unwinding experiments were conducted at room temperature with 25 nM FAM-labeled duplex and 500 nM “trap” 13mer RNA, complementary to the labeled strand, in buffer containing 50 mM Tris (pH 7.8), 120 mM NaCl, 2 mM MgCl_2_, 5% glycerol, 1 mM DTT, and 0.5 units/μL RNasin Plus RNase inhibitor (Promega) [25, 26]. Unwinding reactions with Ded1 (50 nM) were performed in the presence or absence of Xpo1 (550 nM) and Ran[GTP]_Q71L_ (650 nM). Reactions (10 μL) were initiated by the addition of 2 mM ATP and, at the indicated timepoints, stopped by addition of stop solution (5 μL) containing 50mM Tris (pH 7.8), 50mM EDTA, 1mg/mL Proteinase K, 0.6% SDS, 5% glycerol and 0.01% bromophenol blue. Stopped reactions were incubated at room temperature for 20 minutes to allow proteins to be digested by Proteinase K. Samples (3 μL) were separated on 15% native polyacrylamide gels, run at 150 V for 110 minutes at 4°C, and analyzed using a Typhoon 9410 (GE Healthcare). Rates were calculated using single exponential fits to the data, using the equation y = a(1-e^-kt^)+C, where y is the fraction unwound, C is a constant, t is time, a is the amplitude, and k is the rate.

### RNA binding assays

The RNA used for binding experiments was a 25mer FAM-labeled RNA (5’CG AAU UCA AAA CAA AAC AAA ACU AG). The Ded1 proteins (900 nM) were incubated with the FAM-labeled RNA (10 nM) in the presence and absence of Xpo1 (990 nM) and Ran[GTP]_Q71L_ (1.17 μM) in buffer containing 50 mM Tris (pH 7.8), 60 mM NaCl, 2 mM MgCl_2_, 0.1 mg/mL BSA, 5% glycerol, 1 unit/μL RNasin Plus, 0.01% nonidet P-40 (Sigma), and 1mM DTT. Where indicated, the non-hydrolyzable ATP analog adenosine 5′-(β,γ-imido)triphosphate (AMP-PNP) was included at 3 mM concentration. After a 20 min incubation at room temperature, the RNA binding reactions (10 μL) were challenged with competitor RNA solution containing an unlabeled 41mer RNA (27 μM) in 5% glycerol (5 μL), 1 minute before loading on a 7% native polyacrylamide gel [27]. Gels were run at 100V for 80 minutes at 4°C, and analyzed using a Typhoon 9410.

## Results and Discussion

### Stable binding of Ded1 to Xpo1 depends on Ran[GTP] and the Ded1 nuclear export sequence

**Fig 1:**
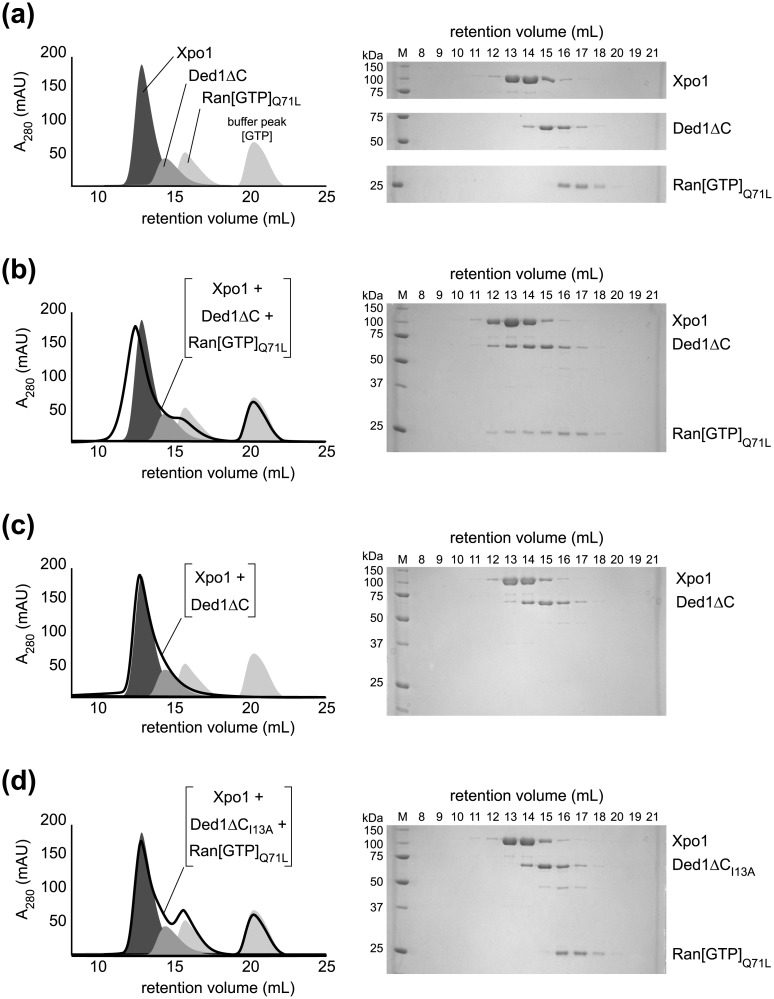
The NES of Ded1ΔC is critical for a Ran[GTP]-dependent interaction with Xpo1. **(a)** The three protein components yield unique profiles by size exclusion chromatography. The profiles for each component applied individually on a Superdex 200 10/300 column are overlaid for comparison: Xpo1 (dark gray), Ded1ΔC (medium gray), and Ran[GTP]_Q71L_ (light gray). The second, larger peak in the Ran[GTP]_Q71L_ profile is excess GTP included in the injected sample. Analysis of each experiment is also shown by SDS-PAGE, illustrating the distribution of peak fractions of each protein in isolation. **(b)** Xpo1, Ded1ΔC and Ran[GTP]_Q71L_ form a complex. The elution profile for Xpo1, Ded1ΔC and Ran[GTP]_Q71L_ co-incubated together before injection is shown as a black trace. The three solid gray peaks of the individual components, as shown in (a), are given for comparison. As shown in the chromatogram and by SDS-PAGE, all three proteins elute in earlier fractions when co-incubated together, consistent with a higher molecular weight complex. **(c)** Ded1ΔC and Xpo1 do not form a stable complex without Ran[GTP]_Q71L_. The elution profile for Ded1ΔC and Xpo1 co-incubated together before injection is shown as a black trace. The three solid gray peaks of the individual components, as shown in (a), are given for comparison. As shown in the chromatogram and by SDS-PAGE, Ded1ΔC and Xpo1 elute at similar volumes as when they are injected individually, suggesting that a stable complex is not formed. **(d)** Ded1ΔC_I13A_ does not form a complex with Xpo1 and Ran[GTP]_Q71L_. The elution profile for Xpo1, Ded1ΔC_I13A_ and Ran[GTP]_Q71L_ co-incubated together before injection is shown as a black trace. The three solid gray peaks of the individual components, as shown in (a), are given for comparison. As shown in the chromatogram and by SDS-PAGE, all three proteins elute at similar volumes as when they are injected individually, indicating that the I13A disruption of the NES interferes with formation of a stable complex. All chromatograms and elution profiles are representative of three or more experiments.

To determine whether the binding of yeast Ded1 to Xpo1 is dependent on Ran[GTP] and the NES of Ded1, we analyzed the association of purified proteins by size exclusion chromatography (SEC). Since high concentrations of full-length Ded1 are poorly behaved in low salt conditions, we used a C-terminally truncated version of Ded1 (residues 1–544, called Ded1ΔC) for co-migration experiments. To maintain yeast Ran in a GTP-bound form, the Q71L variant was used (corresponding to Q69L in human Ran), which is deficient in GTP hydrolysis and has been used by others to stabilize karyopherin:cargo complexes [28]. In the presence of GTP and magnesium, SEC analysis of individual components showed well separated profiles (Fig 1a). When all three protein components were incubated together, a noticeable decrease in retention volume was observed (Fig 1b), suggestive of a Ded1ΔC:Ran[GTP]_Q71L_:Xpo1 ternary complex.

When Ded1ΔC and Xpo1 were incubated together in the absence of Ran[GTP], however, the resulting SEC profile resembled that of the individual components. SDS-PAGE analysis of the fractions revealed that the migration pattern of Ded1ΔC was unaffected by Xpo1 alone (Fig 1c), indicating that the Ded1ΔC:Xpo1 interaction was Ran[GTP] dependent. To determine whether the Ded1ΔC:Xpo1 interaction was also dependent on a nuclear export sequence (NES), we introduced a single amino acid change, I13A, in the N-terminus of Ded1. This I13A substitution was previously demonstrated to disrupt NES-mediated interactions between Xpo1 and other cargo [17]. As shown in Fig 1d, the I13A variant of Ded1ΔC failed to form a ternary complex with Xpo1 and Ran[GTP]. Taken together, these results demonstrate that yeast Ded1 binds to Xpo1 in a manner consistent with the data reported for the *X*. *laevis* ortholog An3 [8, 14], dependent on both Ran[GTP] and an intact nuclear export sequence as observed for canonical cargo of Xpo1. These results further fit with the well-established requirement for the GTP-bound state of Ran and the specific association of leucine rich NESs with Xpo1 for nuclear cargo export [29, 30].

### Analysis of the Ded1ΔC:Ran[GTP]_Q71L_:Xpo1 complex by SAXS

To date, structural information for how cargos bind to Xpo1 is limited to complexes containing snurportin 1 [12, 13] and NES peptides or exogenous NESs fused to snurportin 1 [17]. Unlike other exportins, where cargos directly contact Ran[GTP], snurportin 1 binds Xpo1 at the outer rim of a toroid like structure, away from the centrally located Ran[GTP] binding site. Although multivalent binding allows snurportin 1 to form a complex with Xpo1 in the absence of Ran[GTP], the presence of Ran[GTP] increases affinity of the snurportin NES to associate with Xpo1 [12]. Based on crystal structures, Ran[GTP] assists binding indirectly by facilitating formation of a binding pocket for the aliphatic rich nuclear export sequence typically harbored by Xpo1 cargos [31].

**Fig 2:**
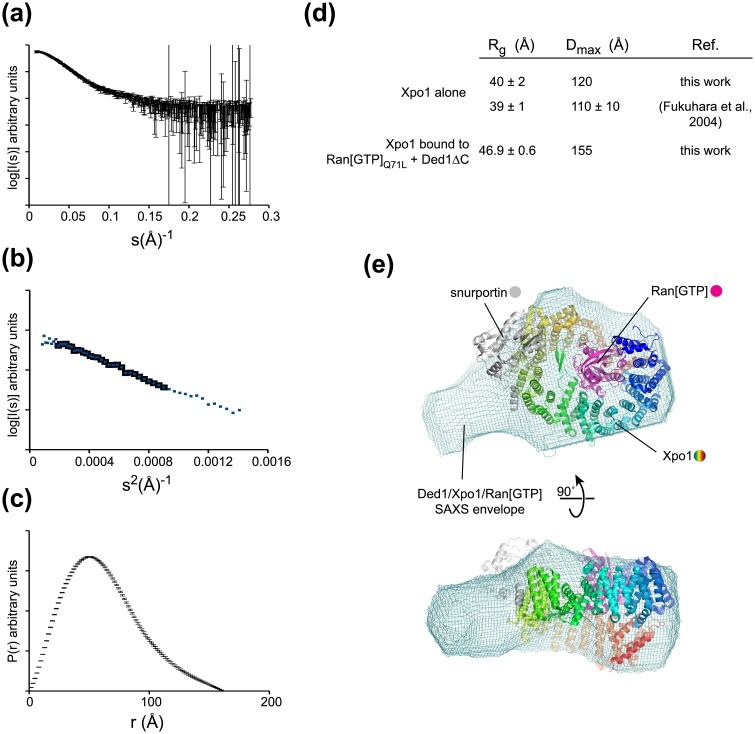
Ded1ΔC binds to the exterior of the Xpo1 toroid. **(a)** Raw scattering data for a purified Xpo1:Ran[GTP]_Q71L_:Ded1ΔC complex. **(b)** Gunier plot for the raw data in (a). The black squares indicate the linear Gunier region. **(c)** P(r) curve for the raw data in (a). **(d)** R_g_ and D_max_ values calculated for Xpo1 alone and in the presence of Ran[GTP]_Q71L_ and Ded1ΔC. Published values are taken from ref. [33]. **(e)** A putative docking of the crystal structure of snurportin 1 bound to Xpo1 and Ran[GTP] (3GJX; [13]) with an averaged, filtered bead model calculated for the Xpo1: Ded1ΔC:Ran[GTP]_Q71L_ complex.

Given the dependence of Ded1 on its NES and Ran[GTP], we set out to determine how Ded1 might interact with Xpo1 using small-angle X-ray scattering (SAXS) (Fig 2). As above, due to aggregation problems of full-length Ded1 protein, we used the Ded1ΔC construct for SAXS experiments. For complex formation, we combined purified Xpo1, Ded1ΔC and Ran[GTP]_Q71L_. We then separated unbound components from the complex by preparative SEC. Fractions containing the ternary complex were collected and concentrated for SAXS experiments. Several dilutions of concentrated samples were used (from 6 mg/mL to 0.65 mg/mL), with the R_g_ remaining consistent for dilutions 0.65 mg/mL to 2.6 mg/mL. Analysis of the linear region of the Gunier plot for these data indicated that samples were free from aggregation (Fig 2b).

Upon Ran[GTP] and cargo binding, Xpo1 undergoes a conformational change [11, 32], which potentially complicates direct comparisons between free Xpo1 and Xpo1-cargo ternary complexes. The R_g_ and D_max_ for Xpo1 alone agreed well with published values [33], while the R_g_ and D_max_ of Xpo1 bound to Ded1 and Ran[GTP] were significantly larger (Fig 2d). To investigate the extent that the Ded1ΔC:Xpo1:Ran[GTP]_Q71L_ complex may resemble the organization observed for the Xpo1:snurportin 1 crystal structure, we used DAMMIF [21] to generate bead models consistent with the SAXS data. These bead model reconstructions for purified ternary complexes demonstrated consistent shapes (normalized spatial discrepancy, NSD = 0.518), and appeared to have a similar organization to the crystal structure of Xpo1 bound to Ran[GTP] and snurportin 1 (Fig 2e and Supplementary Movie 1; [13]). In the Xpo1:Ran[GTP]:snurportin 1 crystal structure complex, Ran[GTP] partially inserts into the center of a toroid formed by the 20 HEAT repeats of Xpo1, leaving a concave bowl-like depression on the other side of Xpo1 [13]. An analogous depression was observed in bead model reconstitutions of our Ded1-containing ternary complexes that likely delimits the interior of the Xpo1 toroid. The bead models also show a protrusion that appears to map to the outer rim of the toroid, similar to the topology of Xpo1:snurportin 1 complexes. As mentioned previously, snurportin 1 is a particularly tight binding cargo and makes additional contacts to Xpo1 outside of its NES. Although the molecular details of the Ded1-Xpo1 interaction are unclear from this low resolution structural data, the SAXS envelopes provide evidence that Ded1 binds at the exterior rather than the central pocket of the Xpo1 toroid. These SAXS data further corroborate the conclusion from the SEC data above that Ded1 interacts with Xpo1 as canonical cargo, through its leucine rich NES and is stabilized by the GTP-bound state of Ran.

### Ded1 ATPase activity is reduced by binding to Xpo1 and Ran[GTP]

**Fig 3:**
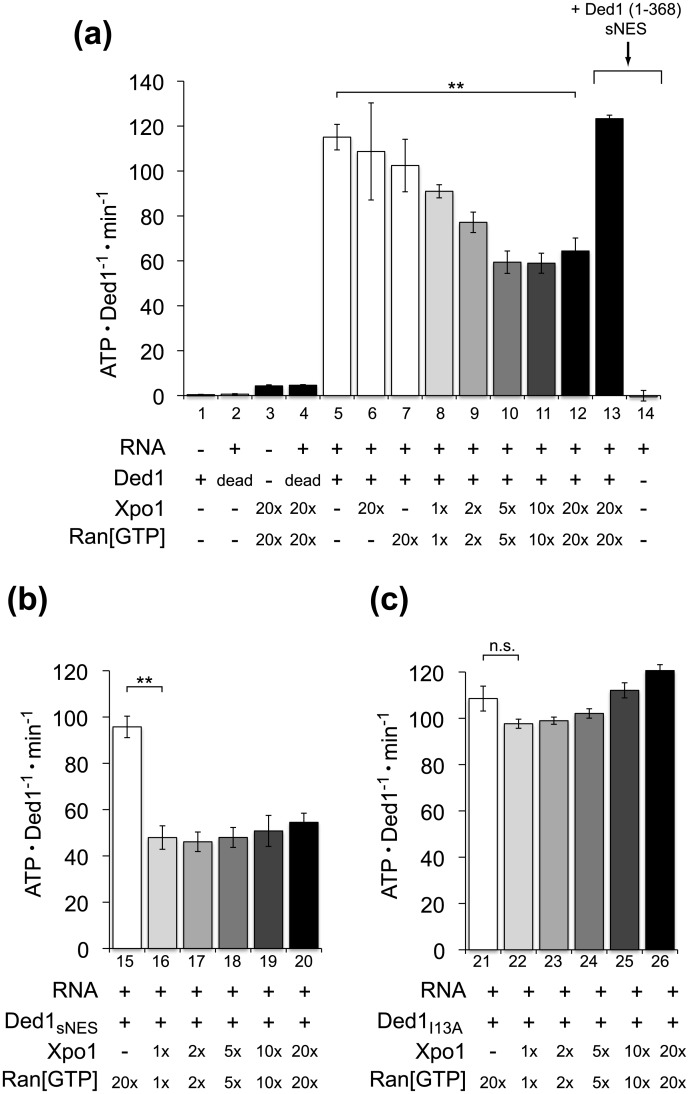
Xpo1 and Ran[GTP] reduce the ATPase activity of Ded1 in an NES-dependent manner. **(a)** The RNA-dependent ATPase activity of Ded1 is reduced in the presence of Xpo1/Ran[GTP]. The rates of ATP hydrolysis for either wildtype (+) or an ATPase-dead variant (Ded1_D306N_) of Ded1 (500 nM) in the presence or absence of yeast whole-cell RNA (320 μg/mL), Xpo1 and/or Ran[GTP]_Q71L_, as indicated. The approximate molar ratios of Xpo1/Ran[GTP]_Q71L_ relative to Ded1 are indicated, corresponding to Xpo1/Ran[GTP]_Q71L_ concentrations of 0.55/0.65 μM (1x), 1.1/1.3 μM (2x), 2.75/3.25 μM (5x), 5.5/6.5 μM (10x), and 11/13 μM (20x). The reaction shown in column 13 additionally contained Ded1(1–368)_sNES_ (12.5 μM), the truncated and ATPase-dead version of Ded1, which relieved inhibition by Xpo1 and Ran[GTP]. **(b)** A tight-binding NES reduced the amount of Xpo1 and Ran[GTP] required for maximal inhibition of Ded1 ATPase activity. RNA-stimulated ATPase activities of Ded1_sNES_ are shown in the absence (column 15) and presence (columns 16–20) of Xpo1 and Ran[GTP]_Q71L_. The concentrations of Xpo1 and Ran[GTP]_Q71L_ are the same as those shown in columns 8–12 of (a). **(c)** Disrupting the NES of Ded1 with the I13A substitution prevents inhibition by Xpo1 and Ran[GTP]. RNA-stimulated ATPase activities of Ded1_I13A_ are shown in the absence (column 21) and presence (columns 22–26) of Xpo1 and Ran[GTP]_Q71L_. The concentrations of Xpo1 and Ran[GTP]_Q71L_ are the same as those shown in columns 8–12 of (a). **P<0.0005; n.s., not significant.

Although it was previously shown that Ded1 orthologs bind to Xpo1, the effect of karyopherin binding on Ded1 activity has not been reported. To investigate if the interaction between Ded1 and Xpo1 modulated Ded1 ATPase activity, we purified full-length Ded1 and conducted ATPase assays in the presence and absence of Xpo1 and Ran[GTP]_Q71L_. Similar to previous observations [24, 34], we observed that yeast whole-cell RNA significantly stimulated ATPase activity of Ded1, with negligible ATPase activity by Ded1 in the absence of RNA (Fig 3a, columns 1 and 5). To ensure that this ATPase stimulation was due to Ded1 and not a result of contamination, we generated an ATPase-dead variant of Ded1, with an Asp→Asn substitution in the Walker B box. As expected, this Ded1_D306N_ variant failed to yield RNA-dependent ATPase activity alone or in the presence of high concentrations of Xpo1 or Ran[GTP] (column 2).

Excess Xpo1 or Ran[GTP] did not significantly alter ATPase activity of Ded1 when added individually (columns 6, 7). However, RNA-dependent ATPase activity of Ded1 was reduced when Xpo1 and Ran[GTP] were both present (columns 8 to 12). At approximately equimolar concentrations of Xpo1 and Ran[GTP], the reduction in activity was small (less than 1.5-fold) but significant. Increasing the molar ratio of Xpo1 and Ran[GTP] further reduced activity up to 2-fold, with saturation apparent at a ~20-fold molar equivalent of Xpo1 and Ran[GTP]. The relatively high concentrations of Xpo1 and Ran[GTP] required to show maximal repression suggested that ATPase inhibition was limited by weak binding of Ded1 with Xpo1 and Ran[GTP]. To test this, we constructed a Ded1 variant with an NES designed to have a high affinity or “supraphysiological” nuclear export sequence (sNES) based on principles outlined by Güttler *et al*. [17], which we refer to as Ded1_sNES_. When ATPase reactions containing Ded1_sNES_ were titrated with increasing amounts of Xpo1 and Ran[GTP], the ATPase activity was reduced to the same extent as wild-type Ded1, approximately 2-fold. However, maximum inhibition was reached at lower Xpo1/Ran[GTP] concentrations with Ded1_sNES_ (Fig 3b), consistent with its predicted higher affinity for Xpo1.

Although ATPase experiments were conducted at concentrations of ATP (5mM) and RNA (320 μg/mL) sufficient to saturate Ded1 alone, high concentrations of excess Xpo1 and Ran[GTP] may have reduced ATPase activity through sequestering RNA substrates away from Ded1. To determine whether Xpo1 and Ran[GTP] could reduce Ded1 ATPase without forming a ternary complex, we tested the Ded1_I13A_ variant, which fails to stably bind to Xpo1 and Ran[GTP] (Fig 1d). In contrast to the wild type Ded1 and Ded1_sNES_ proteins, high concentrations of Ran[GTP] and Xpo1 did not significantly repress the ATPase activity of Ded1_I13A_ (Fig 3c), demonstrating that repression of Ded1 ATPase activity by Xpo1 and Ran[GTP] required an intact Ded1 NES.

While experiments with the Ded1_I13A_ variant demonstrated that Xpo1 and Ran[GTP] did not independently compete with Ded1 for RNA substrates, it was still possible that Xpo1/Ran[GTP] could sequester RNA substrates in a Ded1-dependent manner. As mentioned above, Xpo1 undergoes a conformational change upon binding of cargo and Ran[GTP], which bind cooperatively [11]. It is possible that a cargo-bound conformation of Xpo1 would be required to effectively bind and sequester RNA substrates. That is, although dependent on an intact cargo NES, the reduction in Ded1 ATPase activity could still be indirect through substrate sequestration. Since the Ded1_I13A_ variant is deficient in Xpo1 binding, it would not support a cargo-bound conformation of Xpo1. We therefore tested whether the inhibitory effects of Xpo1/Ran[GTP] could be titrated away with a fragment of Ded1 that should be competent for forming a complex, but should be unable to bind to RNA substrate or hydrolyze ATP. To effectively compete for Xpo1/Ran[GTP] binding, we used a C-terminally truncated protein that only includes the first 368 residues, Ded1(1–368), which excludes the second RecA-like lobe of the ATPase motor and therefore lacks ATPase activity. To ensure that this truncated Ded1 was an effective competitor for Xpo1 binding, the supraphysiological NES was introduced. Compared with Ded1 ATPase activity in the presence of 20-fold molar excess of Xpo1/Ran[GTP], preincubation of the reaction with saturating amounts of Ded1(1–368)_sNES_ yielded ATPase rates similar to that of Ded1 alone (Fig 3a, column 13). Therefore, this N-terminal fragment of Ded1 effectively removed the inhibitory effects of Xpo1/Ran[GTP] on RNA-stimulated Ded1 ATPase activity. These results show that the inhibitory effects of Xpo1/Ran[GTP] on Ded1 arise from formation of the ternary complex, where Ded1 must be physically linked to Xpo1 in order to reduce its ATPase activity.

### Ded1 helicase activity is reduced by Xpo1 and Ran[GTP]

Others have demonstrated that Ded1 unwinds RNA duplexes [34], with a preference for duplexes with single stranded overhangs [35]. Although ATP binding without hydrolysis has been shown to be sufficient for RNA duplex unwinding, ATP hydrolysis is required for enzyme turnover and recycling [27]. Given the reduction in Ded1 ATPase activity in the presence of Xpo1/Ran[GTP], we sought to determine whether helicase activity of Ded1 was similarly inhibited. Since an ~11-fold molar equivalent of Xpo1/Ran[GTP] yielded maximal inhibition of Ded1 ATPase activity, the same molar excesses of Xpo1/Ran[GTP] relative to Ded1 were used in RNA unwinding assays. For the RNA unwinding substrate, an unlabeled 41-mer was annealed to a FAM-labeled 13-mer. To prevent reannealing of the labeled strand, an unlabeled 13-mer RNA oligomer complimentary to the labeled strand was included in excess to trap the labeled strand after melting. After removal of proteins with Proteinase K, annealed and melted substrates were separated by native PAGE.

**Fig 4:**
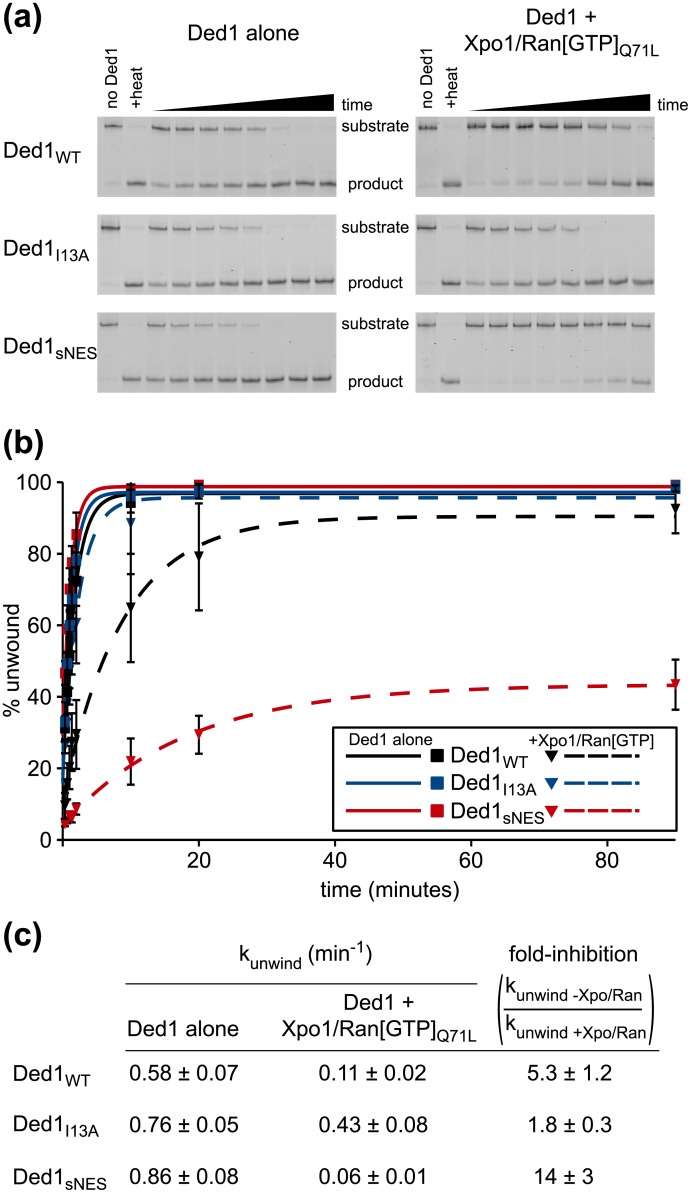
Xpo1 and Ran[GTP] reduce the RNA unwinding activity of Ded1. **(a)** RNA duplexes consisting of a FAM-labeled 13mer annealed to an unlabeled 41mer were incubated with Ded1 variants (50 nM) under unwinding conditions in the presence and absence of Xpo1 (550 nM) and Ran[GTP]_Q71L_ (650 nM). The time points used for each experiment were 0.33, 0.66, 1, 1.33. 2, 10, 20, and 90 min. The reactions were monitored by native PAGE, with a representative gel shown from three independent experiments. **(b)** Quantification of RNA unwinding experiments. Each set of points represents the average of three separate experiments. Single exponential fits are shown for Ded1 variants alone (solid lines) and in the presence of Xpo1/Ran[GTP]_Q71L_ (dotted lines). **(c)** Values for k_unwind_ for data plotted in (b). The reductions in helicase activity upon addition of Xpo1 and Ran[GTP], given as fold-inhibition, are the ratios of k_unwind_ values in the absence and presence of Xpo1 and Ran[GTP].

The trends observed for wild-type, I13A and sNES variants of Ded1 in ATPase assays were mirrored in the unwinding experiments, but were much more pronounced (Fig 4). Despite the presence of an unlabeled 13-mer RNA trap oligonucleotide, not all reactions reached completion at the time points assayed. Other groups have observed similar behavior for crippled DEAD-box helicases or suboptimal unwinding substrates [25, 26]. For wild type Ded1, the presence of excess Xpo1/Ran[GTP] decreased helicase activity ~5-fold. A stronger effect was observed for Ded1_sNES_, with helicase activity reduced ~14-fold with excess Xpo1/Ran[GTP], whereas less than 2-fold inhibition was observed when Xpo1/Ran[GTP] was added to Ded1_I13A_. Similar to the ATPase results, therefore, the influence of Xpo1/Ran[GTP] was NES dependent, indicating that inhibition could be tuned by the strength of the NES-mediated interaction.

### Xpo1 and Ran[GTP] binding dramatically increase the K_M_ of Ded1 for whole cell and poly(A) RNA

**Fig 5:**
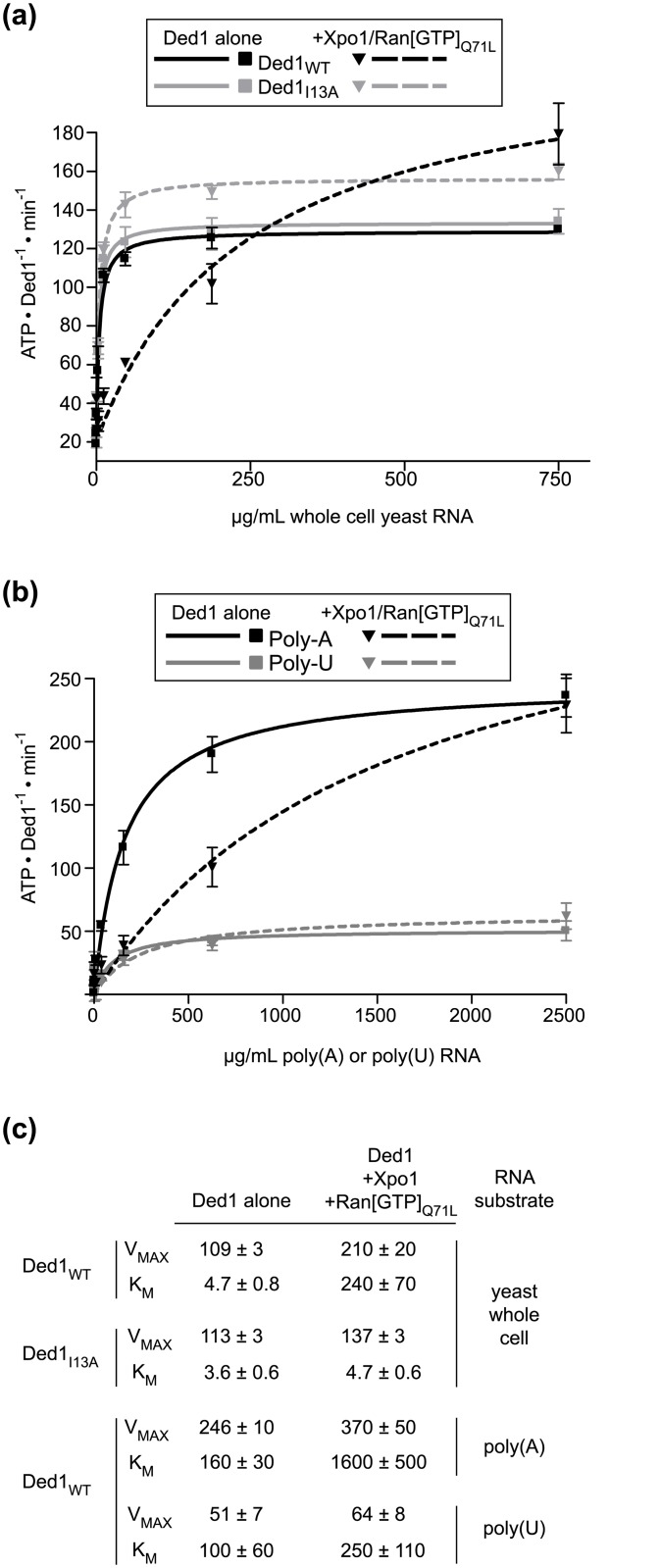
Xpo1 and Ran[GTP] increase the K_M_ of Ded1 for RNA. **(a)** Whole cell yeast RNA titrations in the presence of Xpo1 and Ran[GTP]. Rates of ATP hydrolysis are plotted for 500 nM wild-type Ded1 (black squares) and Ded1_I13A_ (gray squares) in the presence of 0, 0.18, 0.7, 2.9, 11.7, 46.9, 187.5, 750 μg/mL whole cell yeast RNA. The same titration series for wild-type Ded1 (black triangles) and Ded1_I13A_ (gray triangles) was conducted in the presence of 11μM Xpo1 and 13μM Ran[GTP]_Q71L_. Michaelis-Menten fits are plotted for Ded1 (solid black line) and Ded1_I13A_ (solid gray line) alone and with Xpo1/Ran[GTP] (dotted black and gray lines, respectively). **(b)** Michaelis-Menten plots for Ded1 in the absence or presence of Xpo1 and Ran[GTP] using poly(A) or poly(U) RNA substrates. As for (a), ATP hydrolysis rates are plotted for 500 nM Ded1 in the presence of poly(A) (black squares) or poly(U) RNA (gray squares), using RNA concentrations of 0, 0.61, 2.4, 9.8, 39, 156, 625, 2500 μg/mL. The solid and dotted lines show Michaelis-Menton fits. For (a) and (b), the symbols and error bars show the means and standard deviations from three independent experiments. **(c)** Calculated K_M_ and V_max_ values for data plotted in (a) and (b).

To determine whether the reduction in ATPase and helicase activities of Ded1 was the result of reduction in maximal enzyme activity or diminished ability of RNA to saturate the enzyme, we carried out Michaelis-Menten analysis by varying concentration of RNA substrates. As reported previously, saturation with whole cell yeast RNA was reached at concentrations below 75 μg/mL for wild-type Ded1 [36] (Fig 5a). However, when incubated in the presence of a ~10 fold molar excess of Xpo1 and Ran[GTP]_Q71L_, concentrations below 75 μg/mL were insufficient to saturate Ded1 ATPase activity. Instead, we observed a steady increase in ATPase activity, up to a 10-fold higher concentration of whole cell RNA (750 μg/mL). This higher concentration of RNA reflects an approximate 50 fold increase in K_M_ for the Ded1:Xpo1:Ran[GTP]_Q71L_ complex compared to Ded1 alone. In contrast, we observed saturation kinetics similar to wild-type Ded1 for the Ded1_I13A_ variant alone and in presence of the Xpo1 and Ran[GTP] _Q71L_. Because saturation of ATPase activity could not be observed for the Ded1:Xpo1:Ran[GTP] _Q71L_ complex, it is difficult to confidently assign a V_max_ to the ternary complex. However, at high concentrations of whole cell RNA, the ATPase activity of the Ded1:Xpo1:Ran[GTP] _Q71L_ complex was reproducibly higher than Ded1 alone. It appears that binding to Xpo1 and Ran[GTP] reduces the ability of Ded1 to be readily stimulated by mixed substrates, such as those present in whole cell yeast RNA. Because we were unable to observe saturation for the Ded1 ternary complex, it was possible that the Ded1 ternary complex is specifically stimulated by particular RNA species that may be present at low concentrations in whole cell extracted yeast RNA, and the higher K_M_ value reflects limiting concentrations of these species. To test whether limiting concentrations of RNA species in our whole cell RNA samples were responsible for the observed shift in K_M_, we repeated our Michaelis-Menten analysis of Ded1 using single stranded poly(A) and poly(U) RNA substrates (Fig 5b). As reported previously, poly(A), but not poly(U), robustly stimulates Ded1 activity[1] and much higher concentrations of poly(A) were required for maximal stimulation of Ded1 compared to whole cell RNA. As with whole cell RNA, a significant albeit smaller increase in K_M_ was observed for Ded1 in the presence of Xpo1/Ran[GTP]_Q71L_ (Fig 5c), suggesting that the increase in K_M_ for RNA substrates is a general effect of Ded1 binding to Xpo1 and Ran[GTP].

### Stable RNA binding by Ded1 is reduced in the presence of Xpo1/Ran[GTP]

**Fig 6:**
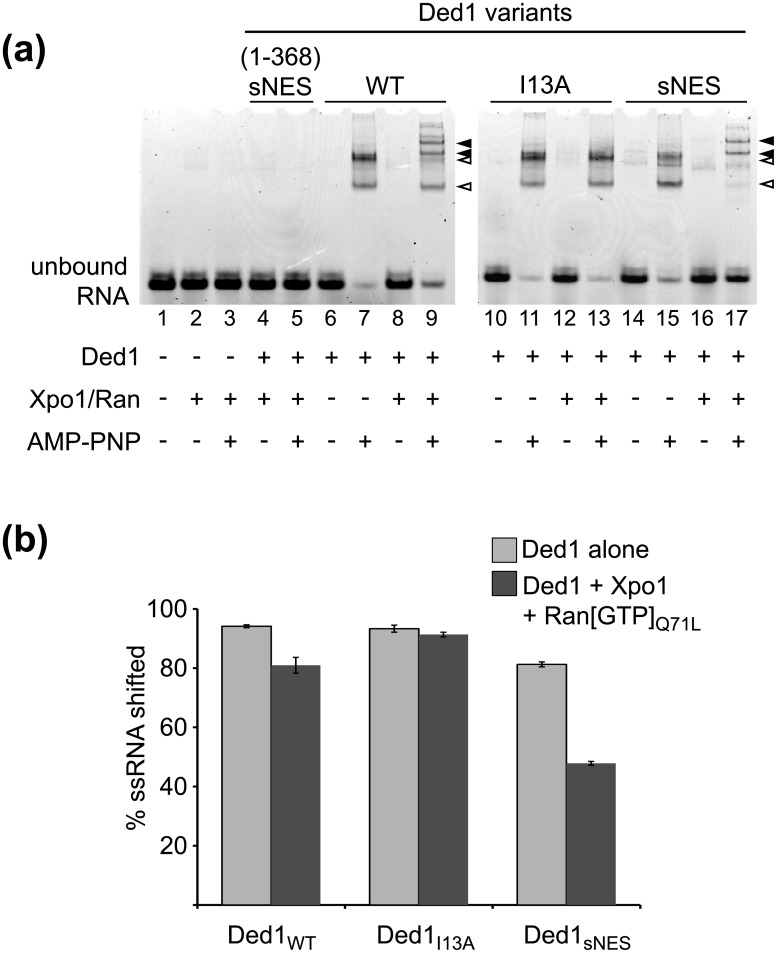
Xpo1 and Ran[GTP] reduce but do not prevent ATP-dependent single-stranded RNA binding activity of Ded1. **(a)** FAM-labeled 25mer RNA oligomers were incubated in the presence and absence of Ded1 variants (900 nM), Xpo1 (990 nM), and Ran[GTP]_Q71L_ (1.17 μM), and resolved by native PAGE after addition of excess unlabeled RNA. Reactions additionally incubated in the presence of the non-hydrolyzable ATP analog AMP-PNP showed super-shifted species, suggestive of protein-RNA complexes. Open triangles indicate shifted RNA species present when Ded1 is incubated with ATP and RNA alone; closed triangles indicate super-shifted RNA species present only in reactions containing Ded1:Xpo1:Ran[GTP] complexes. **(b)** Quantification of the fraction of super-shifted RNA from reactions containing AMP-PNP, full length Ded1 variants, and the presence or absence of Ran[GTP]_Q71L_ and Xpo1. Averages and standard deviations shown are from three independent experiments.

A simple mechanism by which Xpo1:Ran[GTP] might modulate ATPase and helicase activities of Ded1 is by directly effecting the substrate binding: Xpo1 and Ran[GTP] may occlude Ded1 RNA binding sites and reduce stable association of Ded1 with stimulating RNA substrates. To determine if Xpo1 and Ran[GTP] directly occlude the RNA binding surfaces of Ded1, we analyzed ATP-dependent binding to ssRNA with and without Xpo1/Ran[GTP]. To visualize RNA binding by Ded1, a 25mer FAM labeled RNA oligomer was incubated with full length Ded1, and native PAGE was used to separate bound and unbound species (Fig 6). Binding reactions were carried out in the presence of the AMP-PNP, as this nonhydrolyzable ATP analog was previously shown to stabilize Ded1 binding to ssRNA [27]. In order to specifically analyze stable Ded1:ssRNA complexes, the binding reactions were challenged with a 41mer unlabeled RNA oligomer immediately before gel loading. Consistent with previous findings [27, 36–38], in the presence of AMP-PNP, full-length Ded1 produced super-shifted species of the labeled ssRNA, which we interpret to be stable Ded1:ssRNA complexes that remain after challenge from the unlabeled RNA competitor (lane 7). The multiple bands observed in our Ded1:ssRNA binding experiments could be the result of multiple Ded1 molecules binding to a single labeled RNA, and this multiple banding pattern has been observed in Ded1:ssRNA binding experiments from other groups [36–38]. In contrast to the Ded1-containing samples, incubation of labeled ssRNA with Xpo1 and Ran[GTP] without Ded1 did not yield significantly super-shifted RNA species (lanes 2 and 3). Similarly, RNA super-shifting was also not observed when the severely truncated variant Ded1(1–368)_sNES_, lacking the second RecA-like lobe critical for RNA binding, was incubated with Xpo1 and Ran[GTP] (lanes 4 and 5).

RNA super-shifts dependent on Ded1 and AMP-PNP were also observed in the presence of Xpo1/Ran[GTP]. Interestingly, although Ded1_sNES_ alone bound RNA to the same extent as wild-type Ded1 (lane 15), when incubated with Xpo1 and Ran[GTP]_Q71L_ additional super-shifted species were observed that migrated more slowly than bands corresponding to Ded1_sNES_:RNA complexes (compare lanes 15 and 17). In fact, nearly all of the Ded1_sNES_:RNA complexes were converted to these more slowly migrating super-shifted species when incubated with Xpo1 and Ran[GTP]_Q71L_. In contrast, the I13A variant of Ded1 did not yield additional super-shifted species in the presence of Xpo1 and Ran[GTP] (compare lanes 11 and 13). Slower migrating super-shifted species were also observed for wild-type Ded1 when incubated with Xpo1 and Ran[GTP], although unlike the Ded1_sNES_ experiments, bands corresponding to Ded1:RNA complexes were still visible (lane 9). Given the NES-dependent behavior of these slower-migrating species, we interpret these bands to contain Ded1:Xpo1:Ran[GTP]:RNA complexes. The presence of super-shifted Ded1:Xpo1:Ran[GTP] complexes demonstrates that Ded1 is still capable of binding ssRNA in an ATP-dependent manner when bound to Xpo1. We also find that that overall ssRNA binding is reduced with tight association of Ded1 with Xpo1 and Ran[GTP] (Fig 6b). This reduced affinity may partially explain our observation of an increased K_M_ for non-specific substrates in our Michaelis-Menten analysis.

## Conclusions

Exportins such as Xpo1 have a number of functions associated with their nuclear pore trafficking activities. In addition to maintaining nucleo-cytoplasmic gradients, exportins can also prevent aberrant association of binding partners to their cargos [32, 39, 40], and prevent degradation of miRNAs [41]. Here we present evidence that the Xpo1/Ran[GTP] export complex can modulate the activities of the DEAD-box RNA helicase Ded1.

Binding and solution scattering data presented here demonstrate that Ded1 binds canonically to Xpo1 (Figs 1 and 2). We found that this canonical interaction, mediated by an N-terminal NES and Ran[GTP], reduced the helicase activity of Ded1 on a duplex RNA substrate and reduced ATPase activity at moderate concentrations of RNA, while retaining the ability of Ded1 to bind ssRNA.

ATPase repression by Xpo1/Ran[GTP], observed at moderate concentrations of whole cell yeast RNA extract, was sensitive to the nature of the Ded1 NES, which appeared tunable based on affinity (Fig 3). Weakening the Ded1-Xpo1 interaction by using the I13A variant of Ded1, which disrupts the N-terminal NES [17], prevented Xpo1/Ran[GTP] from repressing Ded1. Conversely, outcompeting the wild-type Ded1 NES for access to the NES binding pocket of Xpo1 with Ded1(1–368)_sNES_, an ATPase dead fragment of Ded1, also blocked Xpo1-mediated repression. In agreement with diminishing ATPase activity of Ded1, the presence of Xpo1 and Ran[GTP] also reduced RNA helicase activity of Ded1 (Fig 4). Affinity of the Ded1 NES also correlated with the extent of helicase repression, with the tightest binding Ded1 NES variant having a ~14-fold reduced RNA unwinding activity in the presence of Xpo1 and Ran[GTP].

The apparent repression we observed was not due to a reduced V_max_, but instead an increased K_M_ of Ded1 for RNA in the presence of Xpo1 and Ran[GTP] (Fig 5). Although the final concentrations of RNA substrate needed to saturate ATPase activity of the Ded1:Xpo1:Ran[GTP] complex was much higher than for uncomplexed Ded1, a higher maximal ATPase activity was achieved with the complex compared with Ded1 alone. Consistent with an NES-dependent effect on Ded1 activity, the I13A variant, which has greatly reduced affinity for Xpo-Ran[GTP], displayed maximal ATPase activity at lower RNA concentrations, similar to Ded1 alone.

The increased K_M_ of the Ded1:Xpo1:Ran[GTP] complex for RNA substrates suggests a reduction in affinity for RNA. For the DEAD-box helicases Dbp5 and eIF4A, RNA binding is regulated by Nup214 and PDCD4, respectively, which directly block RNA-interacting surfaces on the helicase domains and inhibit ATPase activity [42–45]. Similarly, Xpo1 may occlude RNA binding motifs of Ded1, either within or outside of the Ded1 helicase domain. However, our RNA binding experiments showed additional super-shifted RNA species consistent with a Ded1-Xpo1/Ran[GTP]-RNA complex, suggesting that Ded1 retains an ability to bind ssRNA in the presence of Xpo1 and Ran[GTP] (Fig 6). Thus, although the apparent affinity of Ded1 for a ssRNA substrate appeared to be reduced in the presence of Xpo1/Ran[GTP], the formation of a complex did not prevent RNA binding. Taken together, we hypothesize that binding to Xpo1 and Ran[GTP] may alter the intrinsic substrate specificity of Ded1.

While substrates of Ded1 in the Xpo1 pathway have not been elucidated, we have demonstrated that like canonical mRNP export factors associated with CBP and eIF4F complexes, Xpo1 is able to modulate Ded1 activity. Xpo1 has been implicated in the export of a variety of RNA subtypes from the nucleus, including ribosomal RNAs [46], snRNAs [30, 47], signal recognition particle RNAs [48–50], as well as viral [9] and cellular mRNAs [51]. The export of mRNAs by Xpo1 is facilitated by protein adaptors that are specific for mRNA subtypes not exported by traditional bulk mRNA export pathways [52, 53]. Ded1 may be acting as one such adaptor, remodeling particular RNA substrates bound by the Ded1:Xpo1:Ran[GTP] complex. Newly exported mRNAs associated with the Ded1:Xpo1 complex would likely be CBP-containing mRNA complexes, before replacement of the CBP by eIF4E during the so called ‘pioneer round’ of translation. By modulating Ded1 activity, Xpo1 would be positioned to act as a regulator of the pioneer round of translation. Xpo1 could act to prevent Ded1 from efficiently remodeling the 5’UTR of the nascent mRNA, thereby reducing efficient translation initiation. Following Ran[GTP] hydrolysis and the release of Ded1 from Xpo1, Ded1 would be free to remodel the 5’UTR, allowing efficient translation initiation. Interestingly, the karyopherins importin-α and importin-β have been proposed to be responsible for exchange of CBP for eIF4E during the pioneer round of translation, linking nuclear transport proteins to mRNP remodeling [54].

The results presented here provide a framework for understanding the influence of NES-mediated interactions on Ded1 activities, and we anticipate future studies that reveal how formation of an Xpo1/Ran[GTP] complex alters Ded1 action.
